# Optimization of anti-tachycardia pacing efficacy through scar-specific delivery and minimization of re-initiation: a virtual study on a cohort of infarcted porcine hearts

**DOI:** 10.1093/europace/euac165

**Published:** 2022-10-05

**Authors:** Shuang Qian, Adam Connolly, Caroline Mendonca-Costa, Fernando Campos, Cristobal Rodero, John Whitaker, Christopher A Rinaldi, Martin J Bishop

**Affiliations:** Department of Biomedical Engineering, School of Imaging Sciences and Biomedical Engineering, Kings College London, London, UK; Invicro, London, UK; Department of Biomedical Engineering, School of Imaging Sciences and Biomedical Engineering, Kings College London, London, UK; Department of Biomedical Engineering, School of Imaging Sciences and Biomedical Engineering, Kings College London, London, UK; Department of Biomedical Engineering, School of Imaging Sciences and Biomedical Engineering, Kings College London, London, UK; Department of Biomedical Engineering, School of Imaging Sciences and Biomedical Engineering, Kings College London, London, UK; Department of Cardiology, Guy’s and St Thomas’ Hospital, London, UK; Department of Biomedical Engineering, School of Imaging Sciences and Biomedical Engineering, Kings College London, London, UK; Department of Cardiology, Guy’s and St Thomas’ Hospital, London, UK; Department of Biomedical Engineering, School of Imaging Sciences and Biomedical Engineering, Kings College London, London, UK

**Keywords:** ȃAnti-tachycardia pacing, Cardiac resynchronization therapy, Implantable cardioverter defibrillator, Patient-specific modelling, Ventricular tachycardia

## Abstract

**Aims:**

Anti-tachycardia pacing (ATP) is a reliable electrotherapy to painlessly terminate ventricular tachycardia (VT). However, ATP is often ineffective, particularly for fast VTs. The efficacy may be enhanced by optimized delivery closer to the re-entrant circuit driving the VT. This study aims to compare ATP efficacy for different delivery locations with respect to the re-entrant circuit, and further optimize ATP by minimizing failure through re-initiation.

**Methods and results:**

Seventy-three sustained VTs were induced in a cohort of seven infarcted porcine ventricular computational models, largely dominated by a single re-entrant pathway. The efficacy of burst ATP delivered from three locations proximal to the re-entrant circuit (septum) and three distal locations (lateral/posterior left ventricle) was compared. Re-initiation episodes were used to develop an algorithm utilizing correlations between successive sensed electrogram morphologies to automatically truncate ATP pulse delivery. Anti-tachycardia pacing was more efficacious at terminating slow compared with fast VTs (65 vs. 46%, *P* = 0.000039). A separate analysis of slow VTs showed that the efficacy was significantly higher when delivered from distal compared with proximal locations (distal 72%, proximal 59%), being reversed for fast VTs (distal 41%, proximal 51%). Application of our early termination detection algorithm (ETDA) accurately detected VT termination in 79% of re-initiated cases, improving the overall efficacy for proximal delivery with delivery inside the critical isthmus (CI) itself being overall most effective.

**Conclusion:**

Anti-tachycardia pacing delivery proximal to the re-entrant circuit is more effective at terminating fast VTs, but less so slow VTs, due to frequent re-initiation. Attenuating re-initiation, through ETDA, increases the efficacy of delivery within the CI for all VTs.

## Graphical Abstract

**Graphical Abstract:**
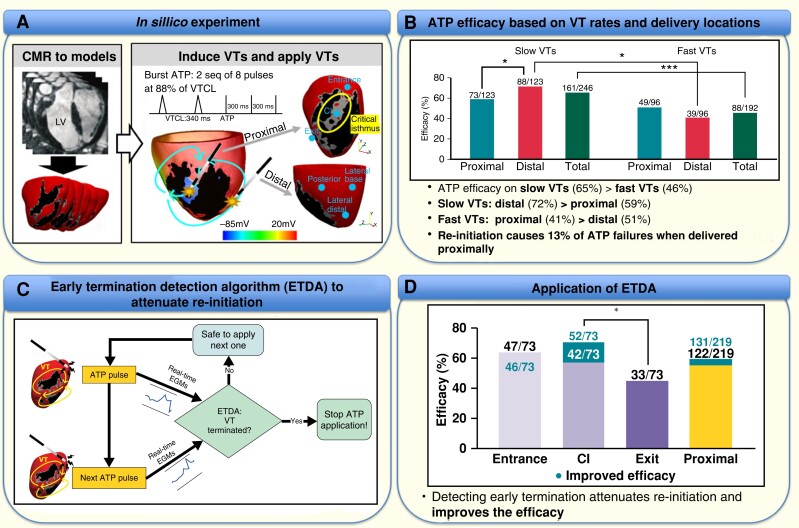
ATP efficacy with respect to VT rates and delivery locations from the re-entrant circuit and ETDA for attenuating re-initiation and improving efficacy. ATP, anti-tachycardia pacing; ETDA, early termination detection algorithm; VT, ventricular tachycardia.

**What’s new?**

- The efficacy of anti-tachycardia pacing (ATP) is dependent on ventricular tachycardia (VT) rates and delivery locations.
- Anti-tachycardia pacing delivery proximal to the circuit is more effective at terminating fast VTs, but less so slow VTs, due to frequent re-initiation.
- Re-initiation may be attenuated by using information from sensed implanted device electrograms to identify early VT termination and subsequently truncate ATP delivery.
- Our automated algorithm not only increases ATP efficacy but also suggests targeting the critical isthmus itself to maximize efficacy.

## Introduction

Ventricular tachycardia (VT) is a potentially lethal cardiac arrhythmia that often degenerates into ventricular fibrillation, leading to sudden cardiac death (SCD). Patients at risk of lethal arrhythmias often receive an implanted electronic device such as an implanted cardioverter defibrillator (ICD) or cardiac resynchronization therapy defibrillator (CRT-D), which detects arrhythmic activity and automatically applies appropriate electrotherapy. Although these devices reduce the risk of SCD and improve mortality, frequent strong biphasic shocks cause long-term myocardial damage that may increase mortality,^1^ with inappropriate shocks resulting in significant pain and psychological issues for device recipients.^2^

In order to reduce the number of defibrillation shocks, current ICDs deploy anti-tachycardia pacing (ATP) to reliably and painlessly terminate VT. Although ATP is known to be effective in terminating slow VTs [VT cycle length (CL) *>* 320 ms], it is less efficient for fast VTs (VTCL: 240–320 ms), with efficacy as low as 50%.^3^ To terminate VT, ATP must interact with the core of the re-entrant circuit, eliminating the excitable gaps that sustain it.^4^ Anti-tachycardia pacing often fails due to an inability of paced wavefronts to reach the re-entrant circuit, due to functional or anatomical barriers.^5^ Thus, it is thought that by varying the pacing locations with respect to the re-entrant substrate, ATP efficacy may be improved.

Previous clinical studies investigating differences in ATP efficacy dependent upon delivery location [left ventricular (LV), right ventricular (RV), or biventricular (Biv) pacing] have failed to reach consensus, suggesting either no differences^6^ or increased efficacy from LV and Biv over RV.^7^ Recently, an *in silico* study^8^ also showed a larger difference (up to 20%) in ATP efficacy when burst pacing was delivered from the LV, compared with the RV. Despite these studies and theoretical considerations,^3^ clear understanding of how ATP efficacy depends upon the proximity of its delivery with respect to the substrate sustaining the VT is lacking. Furthermore, how ‘re-initiation’ (whereby VT is first terminated by the initial ATP pulses, only to then be re-initiated by successive pulses of the ATP sequence) is affected by delivery location also requires further detailed investigation. The importance of such knowledge has been recently enhanced by the widespread use of multipolar cardiac pacing devices (designed for resynchronization therapy), along with state-of-the-art lead-less pacing technology,^9^ providing a clear clinical opportunity for patient-specific ATP delivery configuration and programming.

In this virtual study, we investigated the dependence of ATP efficacy upon delivery location with respect to the re-entrant circuit, applied to a ‘library’ of VT episodes within a cohort of anatomically realistic porcine ventricular infarct models. Standard (ICD, CRT-D) ATP delivery locations were considered, along with optimal ‘scar-specific’ locations, applied to separately analyse both fast and slow VTs. In order to further minimize ATP failures due to re-initiation, we also propose, as a proof-of-concept, an ‘early termination detection algorithm’ (ETDA), utilizing real-time sensed device electrograms (EGMs) to automatically sense early VT termination and cease further ATP pulses.

## Methods

### *In silico* models

**Figure 1:**
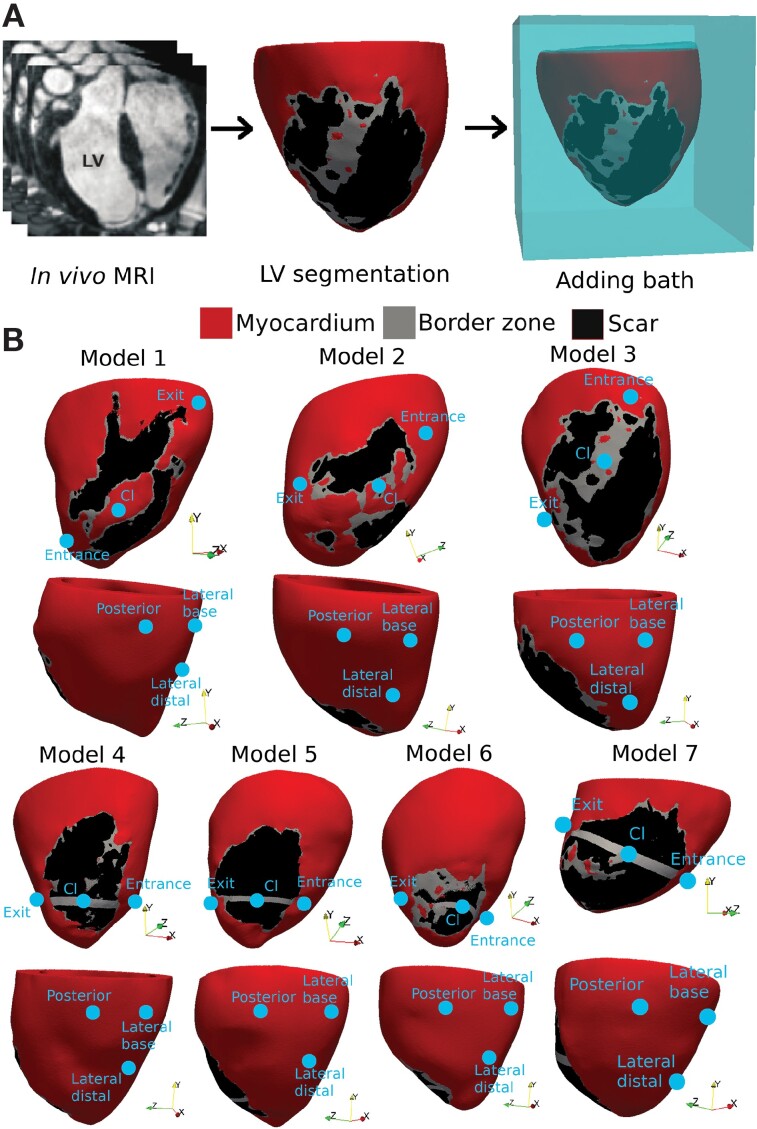
Model generation. (*A*) Pipeline of porcine LV mesh generation from *in vivo* MRI. (*B*) LV models showing six pacing locations. Upper figures show three locations proximal to the scar (RV-septum); lower figures show locations more distal to the circuit (lateral/posterior LV replicating CRT-D devices).

A cohort of seven infarcted porcine LV computational models was constructed directly from *in vivo* cardiac MRI with an isotropic resolution of 1 mm, performed 7 weeks following myocardial infarction, as part of a previous study.^10^ Full details of techniques used in generating the infarct porcine model and constructing the computational models can be found in the Supplementary material online. Compared with the majority of clinical (two-dimensional) CMR scans with slice separations of 8–12 mm, and which often suffer from imaging artefacts due to the presence of implanted devices in pre-ablation patients, this high-resolution three-dimensional pre-clinical data allowed a highly detailed representation of the infarct anatomy, which was critical for capturing the detail and complexity of VT dynamics and its termination by ATP. *Figure 1* shows the model generation pipeline (*Figure 1A*) and seven models (*Figure 1B*). Upon inspection, the original infarct anatomies of Models 4–7 had a large region of compact scar, lacking a critical isthmus (CI) which is essential for inducing and sustaining the VT; consequently, customized CI regions were added to these models to replicate realistic VT pathways, similar to previous computational works.^8^ Animal studies complied with French law and the experimental protocol were approved by the local and national institutional animal care and ethics committee.^10^ More details can be found in the Supplementary material online.

Electrophysiological dynamics was represented by the bidomain model (see Supplementary material online), with simulations performed with the Cardiac Arrhythmia Research Package (https://carpentry.medunigraz.at/).^11^ Porcine cellular ionic dynamics were represented by the Ten Tusscher model,^12^ with ionic properties in the infarct border zone adjusted to produce the required substrate for block and initiation of reentry in the infarcted ventricle, as previously.^8,13,14^

Simulation data of transmembrane potential dynamics in each episode were used to recover the extracellular potential at five typical sensing electrode locations in ICDs/CRT-Ds to give corresponding near-field/far-field EGMs (see Supplementary material online, *Figure S2C*). Sensing vectors included: can-superior vena cava (SVC) coil, can-RV ring, SVC coil-RV ring, RV tip-RV ring, and LV electrode at *LB*-RV tip.^15^

### Ventricular tachycardia induction

**Figure 2:**
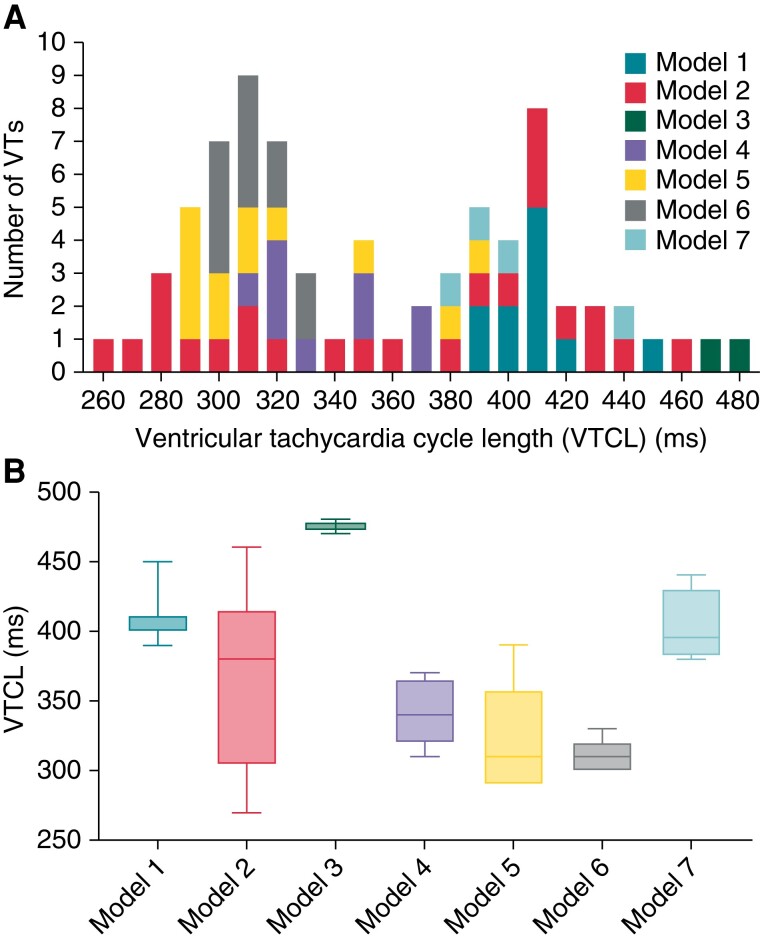
(*A*) Histogram of induced VTCLs in seven porcine models. (*B*) Boxplot of VTCLs of each model. VT, ventricular tachycardia; VTCL, ventricular tachycardia cycle length.

Virtual induction rapid-pacing protocols were applied to all models, with ionic/tissue conductivities varied to augment the induction of multiple VTs with different rates and dynamics (see Supplementary material online, *Section S3*). Note that for each model, in order to induce different VT episodes, tissue ionic properties in BZ were altered from healthy tissue, including a 30–65% reduction of potassium current conductances (*I*_Kr_ and *I*_Ks_) and 10–90% reduction in sodium current conductance (*I*_Na_). See Supplementary material online for full details. In all, 73 monomorphic VTs (MVTs) were induced within the 7 models with VTCLs ranging from 260 to 480 ms (*Figure 2A*). Each episode was simulated for >5 cycles following induction to ensure VT stability prior to applying electrotherapy. Variation in VTCL between models (*Figure 2B*) was due to anatomical and functional differences in the re-entrant circuits defined by the scar substrate, i.e. the CI, as seen in *Figure 1B*. Ventricular tachycardias were categorized based on VTCL as 32 fast VTs (VTCL≤320 ms) and 41 slow VTs (VTCL>320 ms). The activation time maps of example VTs are shown in Supplementary material online, *Figure S1*.

### Anti-tachycardia pacing protocols

Burst ATP was delivered to all VTs at six different locations (constituting 438 virtual scenarios to evaluate efficacy), as shown in *Figure 1B*. The upper figures show three bespoke ‘scar-specific’ locations ‘proximal’ to the CI along the RV septum: the ‘Entrance’ is located where a VT wavefront enters the CI, while ‘Exit’ is where it exits, and the ‘CI’ is located in the middle of CI. The lower images show the other three locations ‘distal’ to the scar, replicating pacing delivered from CRT-Ds, utilizing electrodes on leads typically in the coronary sinus: *Lateral base (LB), Lateral distal (LD),* and *Posterior (Po)*. Details of the location selection are in the Supplementary material online. Burst ATP consisted of one to two sequences of eight pacing pulses with an equal inter-stimulus interval of 88% VTCL,^8^ decreasing by 10 ms in the second sequence. The ATP delivery was initiated at the time reaching 88% of VTCL following the last activation sensed by the ATP-delivering electrode, to ensure delivery to recovered tissue and capture, based on previous work^8^ similar to the setting in current clinically used ICDs. Anti-tachycardia pacing stimuli themselves were delivered via a direct transmembrane stimulus, within the context of our electrophysiological monodomain representation. Such an approach has been shown in a previous study^8^ to accurately replicate the tissue-capturing effect of a more biophysically realistic delivery from the tip of the RV lead within a bidomain representation.

The stimulus current for each ATP pacing was 50 µA/cm^2^, of 5 ms duration.^8^

### Early termination detection algorithm

**Figure 3:**
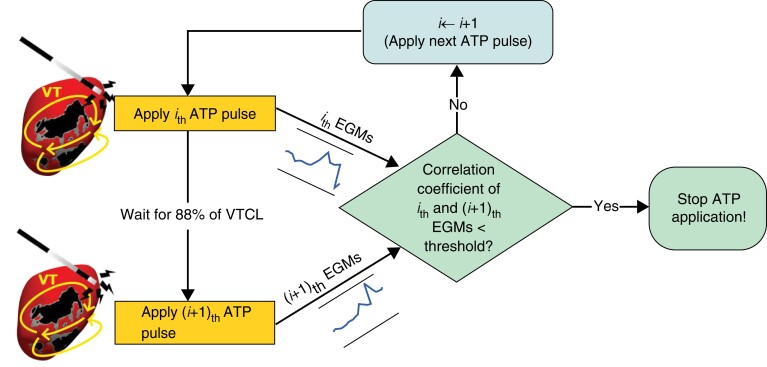
Schematic diagram of ETDA showing the use of sensed EGMs from successive ATP pulses to determine VT termination and truncation of subsequent ATP. ATP, anti-tachycardia pacing; VT, ventricular tachycardia.

**Figure 4:**
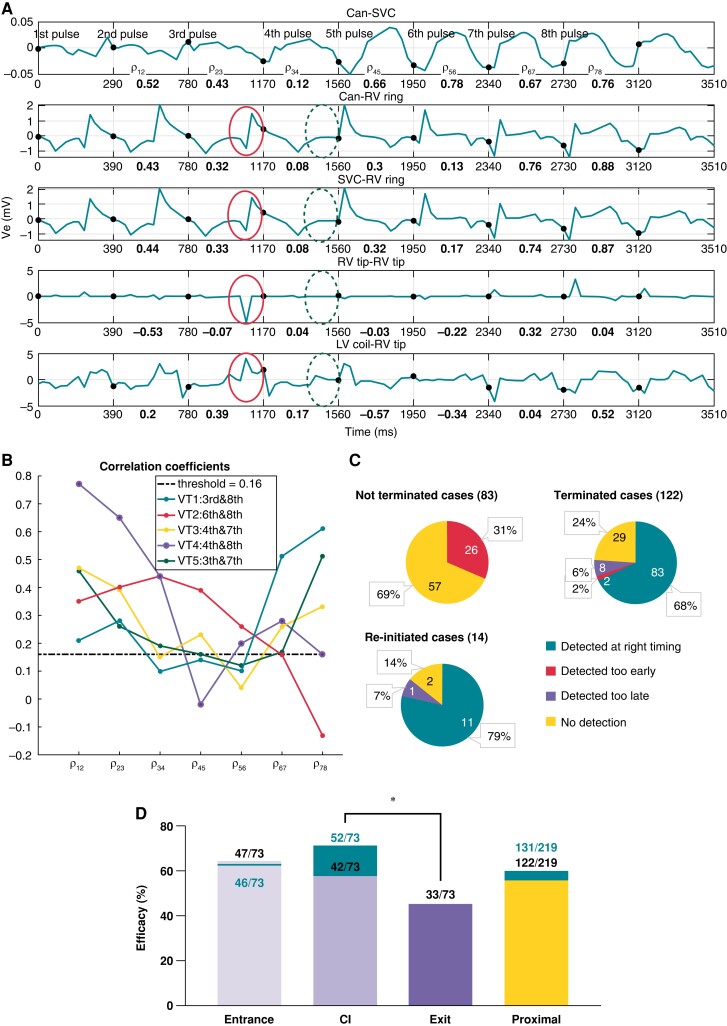
Early termination detection algorithm description. (*A*) EGMs measured during re-initiation shown in *Figure 7* with corresponding CCs in bold. Solid/dashed circles mark morphological differences in EGMs before/after VT termination. (*B*) Mean CCs between successive segments of EGMs during five ATP re-initiation cases. Legend details ATP pulse causing VT termination and re-initiation. (*C*) Early termination detection algorithm performance during proximal (RV-septum) delivery for not terminated, terminated, and re-initiation scenarios. (*D*) Enhanced ATP efficacy by ETDA. For ATP proximal delivery after applying ETDA separately analysed for fast and slow VTs as in orange. Anti-tachycardia pacing efficacy without ETDA is the same as that shown in *Figure 5*. *P < 0.05.

We proposed a proof-of-concept algorithm, termed ETDA (*Figure 3*), which utilizes sensed EGMs (from ICDs/CRT-Ds electrodes) during ATP, to identify initial termination and stop the further application of pulses, thus preventing re-initiation. First, each sensed EGM (*Figure 4A*) is discretized into time segments, based on the VTCL. Correlation coefficients (CCs) between all neighbouring time segments are calculated, and averaged over all five measured EGMs, in an attempt to identify sudden changes in EGM morphology which may indicate termination. A discriminating threshold on the CCs was chosen by comparing all re-initiation cases with the actual VT termination time observed from simulation results. Finally, ETDA was applied to ATP cases, and ATP efficacy was re-assessed.

### Statistical analysis

The ATP efficacy between different pacing locations was compared with Pearson’s *χ*^2^ test^16^ for independence for multiple independent group comparison with binary data, with *P* < 0.05 considered significant.

## Results

### Anti-tachycardia pacing efficacy relative to delivery location: all ventricular tachycardia cycle lengths

Burst ATP was delivered from six locations (three proximal, three distal to circuit) to all 73 induced VTs. Approximately a 20% increase in efficacy was seen between the application of one and two ATP sequences, across all delivery locations (see Supplementary material online, *Figure S3*).

**Figure 5:**
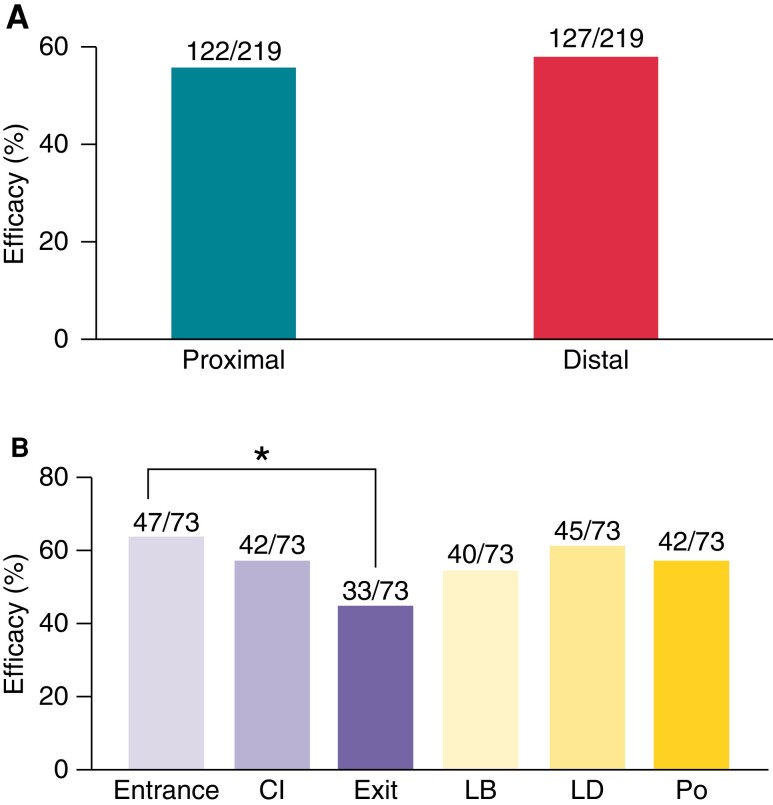
Comparsion of ATP efficacy delivered from different locations. (*A*) Total ATP efficacy delivered from locations proximal and distal to the re-entrant circuit. (*B*) Anti-tachycardia pacing efficacy of all locations including three proximal locations: Entrance, CI, and Exit and three distal locations: Lateral base (LB), Lateral distal (LD), and Posterior (Po).

As shown in *Figure 5*, the overall efficacy of two sequences ATP delivered from locations proximal to the scar (near RV septum) was 56%, being similar to the efficacy delivered from locations distal to the scar (CRT-D locations, 58%) (*P* = 0.63).

Separate analysis of ATP efficacy delivered from the three proximal (*Entrance*, *CI*, *Exit*) and the three distal locations (*LD, LB, Po*) was then performed. Within proximal locations (*Figure 5*), efficacy was significantly higher when applied at the *Entrance* (64%) compared with the *Exit* (45%) (*P* = 0.02), with CI efficacy being mid-way between the two (58%). In contrast, within distal locations, efficacy was similar between the three CRT-D locations (ranging from 55 to 62%).

### Anti-tachycardia pacing efficacy for different ventricular tachycardia rates at specific delivery locations

**Figure 6:**
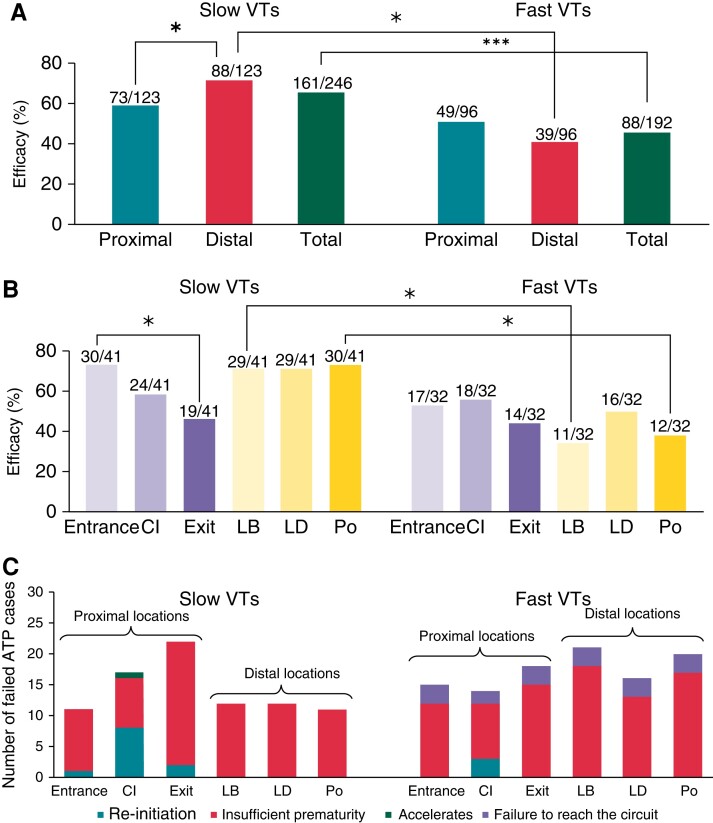
Anti-tachycardia pacing efficacy regarding VT rates and ATP failures. (*A*) Efficacy of ATP proximal and distal delivery and the total for fast and slow VTs. (*B*) Anti-tachycardia pacing efficacy of all locations is similar to that shown in *Figure 5*. (*C*) Mechanisms for failed ATP cases in fast and slow VTs. Location nomenclature as in *Figure 5*. * *P* < 0.05, ***P < 0.0001. VT, ventricular tachycardia.

As shown in *Figure 6*, separate analysis of ATP efficacy based on VTCL reveals that ATP delivered from all locations was significantly more efficient for slow VTs than for fast VTs (65 vs. 46%, *P* = 0.000039). Interestingly, when separately considering slow and fast VTs (*Figure 6*), the efficacy of ATP delivery from proximal locations was similar (59 vs. 51%, respectively, *P* = 0.22). However, in the case of delivery from distal locations, ATP was significantly more efficacious for slow VTs (72%) than for fast VTs (41%) (*P* < 0.00001).

### Anti-tachycardia pacing efficacy for different delivery locations for specific ventricular tachycardia rates

**Figure 7:**
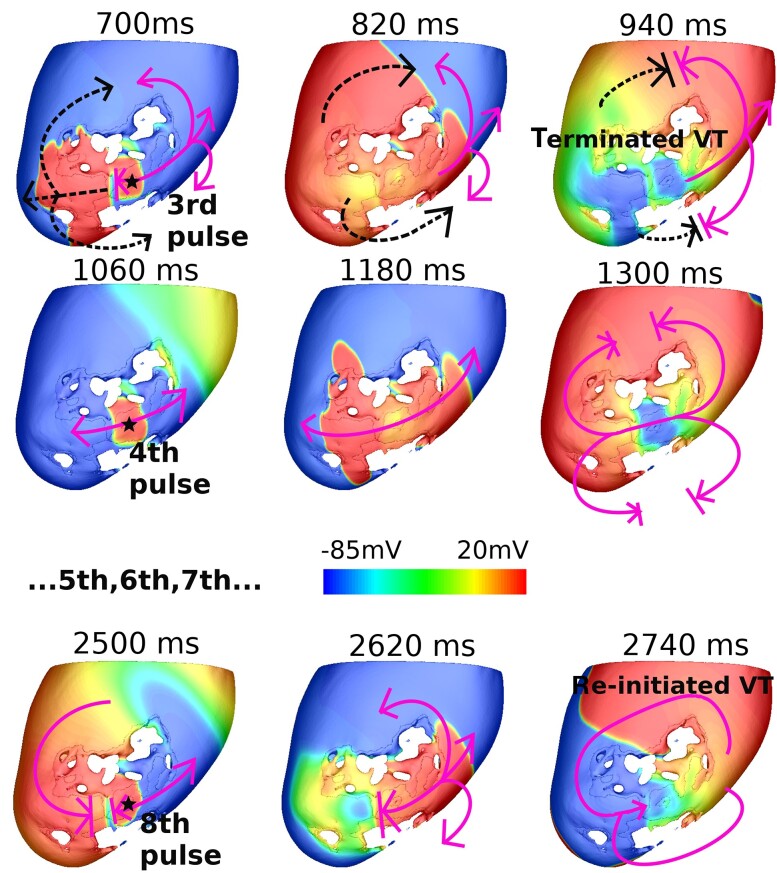
*V*_m_ maps of an example re-initiation which applied at the CI in Model 2. Star shows ATP delivery location; dashed arrows show VT propagation; solid arrows show ATP propagation. Ventricular tachycardia first terminated by third pulse (700 ms), then re-initiated by eighth pulse (2500 ms).

For the separate groups of slow and fast VTs, we now assess potential differences in efficacy based on delivery location. As shown in *Figure 6*, in the case of slow VTs, ATP delivered from more distal locations was significantly more effective compared with delivery from proximal locations (72 vs. 59%, *P* = 0.044). Although more successful overall, there was relatively little difference in efficacy between different distal location delivery (*LB, LD, Po*), 71–73%. In contrast, there was an important difference in efficacy between specific proximal delivery locations, with delivery from *Entrance* being significantly more efficacious than from the *Exit* (73 vs. 46%, *P* = 0.013) (*Figure 7*).

Opposite to the trend for slow VTs, in the case of fast VTs, delivery from distal locations was less effective compared with proximal locations (41 vs. 51%, *P* = 0.148) (*Figure 6*). As shown in *Figure 6*, no significant differences in efficacy were seen within proximal (44–56%) and distal (34–50%) location delivery locations.

### Anti-tachycardia pacing failure mechanisms

The mechanisms responsible for all failed ATP cases were determined by a detailed inspection of simulations results (*Figure 6*). In both slow and fast VTs, ATP primary fails due to insufficient prematurity—see Supplementary material online, *Figure S4* for a detailed example. Re-initiation is a less common failure mechanism, found in 11 slow VT cases and 3 fast VT cases (accounting for 6.4% in total), which was only seen when delivered from locations proximal to the circuit; an example of which is shown in *Figure 7*. Here, the VT is initially terminated by the third pulse (solid arrows, 700 ms). Subsequently, Pulses 4–7 cause benign orthodromic and antidromic wavefronts which collide (1060–1300 ms). However, progressive conduction slowing means that the orthodromic wavefront from the eighth pulse is eventually blocked within the CI (2500 ms) by refractory tissue from the early pulse. Consequently, a new VT with opposite chirality is induced by the (unblocked) antidromic wavefront (2740 ms).

Only in fast VTs, ATP failure also occurred due to failure to reach the circuit. Detailed analysis of other mechanisms of failure (acceleration) is in Supplementary material online, *Figure S5*.

### Early termination detection algorithm for identifying re-initiation

Due to the identified high rate of re-initiation for ATP delivered proximal to the circuit, we evaluated the effectiveness of our novel ETDA approach to detect VT termination. *Figure 4* shows the five measured EGMs during the case of re-initiation from *Figure 7*. The corresponding CCs, which quantify the similarity between successive segments of the EGMs (each pulse), are shown in bold. As shown in *Figure 7*, the VT is terminated after the third pulse and re-initiated after the eighth pulse. Noticeable differences in specific aspects of EGM morphologies become apparent between the third and fourth segments (marked in solid and dashed circles, respectively, in *Figure 4*). These differences in EGM morphologies are also reflected in the corresponding CCs between segments, which are all ≤0.17 at this time.

*Figure 4* shows the temporal change in the mean CCs between segments over all sensed EGMs during ATP application in five examples of re-initiation. The CCs are seen to sharply decrease just before application of the pulse resulting in VT termination. All correlation CCs dropped below the threshold of 0.16 just after the terminating pulse, or one pulse later.

### Application of early termination detection algorithm to improve anti-tachycardia pacing efficacy when pacing proximal to the circuit

Re-initiation was only found to cause ATP failure during application from locations proximal to the circuit (*Figure 6*); thus, ETDA was applied to all applications delivered from those locations only. Overall, ETDA correctly identifies the time of termination in 79% of re-initiation cases and also in 68% of all other cases (*Figure 4*). A detailed analysis of the performance of ETDA can be found in the Supplementary material online.

Applying ETDA to all proximal locations was seen to improve the overall ATP efficacy from 56 to 60% (*Figure 4*). The efficacy of ATP delivered at the *CI* was improved most from 58 to 71% (*P* = 0.084). As a result, this improved ATP efficacy delivered from the *CI* (71%) resulting in its efficacy now being significantly higher than delivered from the *Exit* (45%, *P* = 0.0014), but less significantly higher than from *Entrance* (45%, *P* = 0.29). Analysis based on VTCL further showed that ETDA application improves ATP efficacy delivered at the *CI* for slow VTs from 59 to 76%, and for fast VTs from 56 to 66%; thus, *CI* application now becomes the highest efficacy for all VT rates.

## Discussion

The main finding from this *in silico* study was that, in case of slow VTs, ATP delivered *distal* to the scar is significantly more efficacious than delivery *proximal* to the scar; however, for fast VTs, delivering ATP *proximal* to scar is more effective than distal delivery. Anti-tachycardia pacing efficacy also varies depending upon specific proximal/distal delivery location; for example, in slow VT cases, delivering at the *Exit* site of VT is significantly less efficient than delivering at the *Entrance* site, with a similar (although non-significant) trend also being seen for fast VTs. Through detailed mechanistic analysis of failed ATP cases, we uncovered that ATP delivered from locations proximal to the re-entrant circuit (along the RV-septum) was more likely to cause re-initiation than delivery further from the circuit (applied at the lateral/posterior LV wall through a CRT-D LV lead). We showed that the efficacy of proximal delivery can be improved through the use of a novel algorithm (ETDA) which automatically processes device-sensed EGMs to detect signs of early termination, truncating the ATP sequence, reducing re-initiation. Attenuating re-initiation during ATP delivery increases efficacy for delivery proximal to the re-entrant circuit for both slow and fast VTs, where the most significant improvement is seen when delivered within the *CI*.

### Anti-tachycardia pacing delivery within the critical isthmus is more effective for fast ventricular tachycardias

Recent clinical studies have suggested that ATP efficacy may be improved when delivering closer to the site of the re-entrant circuit; for example, ATP is more successfully delivered from the LV (where the scar is predominantly located) above RV pacing,^7^ whilst ATP is more efficient in treating VTs with apical exit sites than basal exit sites.^17^ However, performing controlled clinical experiments to further investigate this is challenging due to patient variability of scar anatomies along with exact knowledge regarding electrode placement relative to scar. The use of computational models allows us to investigate this potentially important issue in a detailed and highly controlled environment.

In this study, the initial analysis considering all VT episodes suggested that applying ATP proximal to the re-entrant circuit (at RV-septum locations, similar to RV pacing electrodes in conventional ICDs) achieves similar efficacy as delivering more distal to the circuit at the lateral LV wall (from epicardial locations potentially accessed by multipolar CRT-D electrodes in the LV veins) (*Figure 5*). Dichotomizing VT episodes into slow and fast VTs, however, revealed that for slow VTs, ATP application distal to the circuit was more effective than proximal. Detailed analysis of the mechanisms of failure (*Figure 6*) revealed that an important reason for ATP failure in fast VTs is ‘failure to reach the circuit’, not present in slow VTs (and, in fact, only found in very fast VTs with VTCL 260–280 ms). This is consistent with the fact that faster VTCL leads to small excitable gaps, so it is harder for ATP pacing waves to interact and stop the VT. Proximal ATP delivery (i.e. pacing very close/within the circuit itself) may attenuate this mechanism of failure, which may be what drives the increased efficacy of proximal over distal delivery in the case of fast VTs.

Individual analysis of specific proximal and distal locations based on VTCL highlighted that for all proximal locations delivered to slow VTs, in contrary to theoretical considerations,^3^ ATP application at the *Exit* site of the VT was found to be significantly less effective than application to the *Entrance* site. Thus, directly accessing the CI via the orthodromic wavefront (at the *Entrance*), as opposed to the antidromic wavefront (via the *Exit*), was more effective at terminating VTs.

### Re-initiation drives a higher failure rate when pacing proximal to circuit

The conflicting findings regarding ATP efficacy for proximal vs. distal delivery locations between fast and slow VTs, described above, can be further explained by analysing the ATP failure mechanism (*Figure 6*). For slow VTs, no cases failed due to re-initiation (or acceleration) when pacing from distal locations, driving down the lower failure rate compared with proximal locations. This suggests it may be safer to pace further from the re-entrant circuit for slow VTs, particularly as re-initiation may change the morphology of the VT, or decrease the VTCL making it harder to subsequently terminate. However, for fast VTs, re-initiation (and acceleration) was largely not observed, which may be an additional reason driving down the overall failure rate of proximal delivery locations, showing it now to be more efficacious relative to distal delivery locations.

Detailed analysis of cases of ATP failure (*Figure 6*) highlighted that the failure mechanism of re-initiation was only observed when delivered from locations proximal to the circuit (RV-septum locations), but not when pacing distally (CRT-D locations). More specifically, re-initiation (whereby the VT is first terminated by several successive ATP pulses, only to be re-induced following subsequent unidirectional block of later pulses—*Figure 7*) was most frequently seen when ATP was delivered within the CI itself (11/14 of all re-initiation cases). Conduction of a wavefront within the CI is known to be less robust and susceptible to block than in remote regions of the myocardium, due to electrophysiological and structural remodelling. For example, block is particularly likely at the isthmus mouth where electrotonic loading source–sink mismatches may occur, although unidirectional block is also likely *within* the isthmus itself due to the cable-like nature, naturally preventing propagation normal to the isthmus axis. Thus, in current clinical devices, applying ATP from sites proximal, or within, the re-entrant circuit, may in fact be less efficacious for slower VTs, driven by this higher likelihood of re-initiation, which counteracts any benefit of intrinsically successfully penetrating the circuit. In the case of fast VTs, the smaller excitable gap means that block of both the antidromic and orthodromic wavefronts is likely, meaning the required unidirectional block for re-initiation occurs less readily, and re-initiation is less problematic.

### Optimized anti-tachycardia pacing through attenuating re-initiation through early termination detection algorithm

The current ICD/CRT-D devices constantly monitor near-field/far-field EGMs using the sensing electrodes that form part of the devices. Here, we introduce a proof-of-concept algorithm (ETDA) which has the potential of using sensed EGMs to automatically detect VT termination by early ATP pulses, truncating the subsequent sequence, to improve the efficacy by preventing re-initiation. By using ETDA, 79% of re-initiation cases in this cohort were successfully detected (*Figure 4*), allowing ATP to be stopped before VT re-initiates. After applying ETDA during the simulated ATP protocols, we found the total ATP efficacy from proximal delivery locations was improved, particularly from the *CI* location, which became the most efficient pacing location across all scenarios (*Figures 4* and *5*).

Our presented ETDA represents a proof-of-concept approach and provides undoubtable scope for further optimization of the specific algorithm. In particular, if the VT is entrained or reset, then fusion will occur during ATP, leading to a (potentially distinct) difference in sensed EGM morphology, in the absence of VT termination. Nonetheless, these initial results demonstrate the important concept of optimizing ATP by minimizing re-initiation in real time. Although re-initiation is not the major mechanism for ATP failure, it has been commonly seen in other computational and clinical studies;^8,18^ thus its attenuation through advanced ICD-programming may provide an important clinical overall increase in the efficacy of this electrotherapy. Moreover, it is commonly found in both our simulations (see Supplementary material online, *Figure S3*) and the literature^8^ that adding more pulses/sequences could reduce ATP failure due to insufficient prematurity, thus improving ATP efficacy. Given our ETDA demonstrates the potential to identify VT termination in real time (or lack thereof), such an approach could also see utility by optimizing the number of ATP pulses/sequences applied.

### Clinical implications of our proposed anti-tachycardia pacing optimization strategy

Theoretical analysis suggests that delivering ATP from regions closer to the circuit should increase the likelihood of penetrating the excitable gap, terminating the VT.^3^ We have shown that delivering ATP from sites close to the CI of the scar reduces a primary reason for ATP failure in the case of fast VTs (failure to reach the circuit), increasing efficacy relative to sites further from the scar. However, in the case of slow VTs, ATP efficacy is actually less when applied proximal to the scar, because of the higher rate of re-initiation seen. This raises the possibility that real-time alteration in applied ATP delivery location choice in a multipolar device, based on the sensed VT rate by the device prior to therapy delivery, may be beneficial.

However, should the risk of re-initiation be attenuated through an approach such as ETDA, this may suggest targeting the CI itself in a patient-/scar-specific manner is beneficial. Specifically, with recent advances in pre-implant clinical cardiac scar imaging, which can reliably identify scar anatomy, as well as potential isthmuses,^19^ it may be possible to guide the specific implantation locations ICD/CRT-D leads. The recent emergence of multipolar cardiac pacing devices for CRT-D, along with state-of-the-art lead-less pacing technology,^9^ also suggests the practical possibility of delivering ATP from different electrode locations in a flexible manner, potentially also incorporating our novel ETDA to further optimize device performance and programming. Further technological advances in the use of EGM information to locate the sites of origin of VTs^15^ also suggest the possibility of real-time decision-making on the optimal electrode to deliver ATP.

### Limitations and future work

The infarcted porcine experimental models used to create the computational model cohort used here were all anteroseptal scars. Although VT morphologies may be expected to be similar for different scar locations within the ventricle, the ensuing circuits may be at different distances relative to the RV-septum and lateral LV wall ATP delivery sites tested here. Nonetheless, splitting our analysis into proximal and distal delivery may be applied to VT substrates in different locations, which may be specifically targeted in a similar manner with lead-less pacing technologies.

Furthermore, in order to expand our cohort to replicate as many as possible scar geometries as the substrate of VT induction, in four models, we artificially introduced pathways through the scar in order to provide a viable re-entrant circuit. It is possible that such idealized substrates may lead to less realistic (and less arrhythmogenic) scars that might impact our main findings. Therefore, we conducted a subcohort analysis of ATP efficacy using only the three realistic (unmodified) infarcted models (with slightly more complex re-entrant pathways through the infarct), as shown in the Supplementary material online. Such subanalysis revealed that the trend of ATP efficacy for different VT rates pacing proximally and distally remained unchanged, compared with the whole cohort analysis, although the results in this subanalysis were not all statistically significant.

Our computational models do not include representations of the Purkinje system (PS), which may indeed lead to more complex activations, both of the VT itself and the dynamics of ATP delivery. However, other previous works, which also lacked representation of PS, have shown important clinical utility in guiding the development of novel ATP algorithms.^8^ In addition, our models did not include the RV which may limit its ability to replicate the complex VT patterns through the RV. However, the overall efficacy (56%) in terminating all VTs using burst ATP electrotherapy (with 0.23% acceleration) found in our models is closely comparable to clinical observations and other computational studies using full biventricular models.^3,8^ Despite the success of our ETDA approach, other methods of representing the EGM sensing electrodes and obtaining extracellular potentials may also be investigated (full/pseudo-bidomain, lead-field), which may lead to slight morphological changes to the recovered EGM signals and may further enhance the discriminatory power of our algorithm. Finally, the findings presented here require further validation in pre-clinical and clinical contexts, for which the work here provides important insights for the focus of such follow-on studies.

## Conclusions

Delivering ATP from sites proximal to the scar enhances ATP efficacy in fast VTs, but decreases efficacy in slow VTs, partly driven by an increased incidence of re-initiation when ATP is delivered closer to the *CI* sustaining the VT. The EGMs measured from standard ICD/CRT-D devices may be utilized to attenuate re-initiation through early detection of VT termination and subsequent truncation of delivered ATP pulses.

## Supplementary Material

euac165_Supplementary_Data

## Data Availability

The data underlying this article will be shared on reasonable request to the corresponding author. In addition, the cohort of infarcted porcine ventricular models is also available in this article.^20^
