# Supplementary material for: Optimization of anti-tachycardia pacing efficacy through scar-specific delivery and minimization of re-initiation: a virtual study on a cohort of infarcted porcine hearts
Source: Europace. 2022 Oct 5;25(2):716–25. doi: 10.1093/europace/euac165 (PMC9935023; doi:10.1093/europace/euac165)
Supplement: euac165_Supplementary_Data [file euac165_supplementary_data.zip › Data availability Statement.docx]

Data availability Statement

The data underlying this article will be shared on reasonable request to the corresponding author. In addition, the cohort of infarcted porcine ventricular models is also available in this article:

Whitaker J, Neji R, Kim S, Connolly A, Aubriot T, Calvo JJ, et al. Late Gadolinium Enhancement Cardiovascular Magnetic Resonance Assessment of Substrate for Ventricular Tachycardia With Hemodynamic Compromise. Front Cardiovasc Med. 2021;8(October):744779.
