# Supplementary material for: Optimization of anti-tachycardia pacing efficacy through scar-specific delivery and minimization of re-initiation: a virtual study on a cohort of infarcted porcine hearts
Source: Europace. 2022 Oct 5;25(2):716–25. doi: 10.1093/europace/euac165 (PMC9935023; doi:10.1093/europace/euac165)
Supplement: euac165_Supplementary_Data [file euac165_supplementary_data.zip › QS_ATP_supple_revised_18July22.docx]

Optimisation of anti-tachycardia pacing efficacy through scar-specific delivery and minimisation of re-initiation: a virtual study on a cohort of infarcted porcine hearts

Shuang Qian, PhD,* Adam Connolly^‡^, PhD, Caroline Mendonca-Costa, PhD,* Fernando Campos, PhD,* Cristobal Rodero, Msc,* John Whitaker, PhD,*^§^ Christopher A. Rinaldi, MD, FHRS,*^§^ and Martin J. Bishop, PhD*

Supplemental material

1.Generation of porcine models and construction of computational models

The pipeline used for model construction of a cohort of 7 infarcted porcine LV is described here, as shown in Figure 1A. Late-gadolinium enhanced MRI was performed seven weeks following myocardial infarction. The technique of generating the porcine infarct experimental-preparation was developed by ^1^ and was demonstrated to have similar LV remodelling and arrhythmia mechanisms to human post-infarct VT, specifically with the healed infarct resembling the human pathophysiology with subendocardial arrhythmia origin and characteristics of nonuniform anisotropic conduction. The same procedure was adopted by our own team in other studies where this pre-clinical model was further analysed ^2,3^, and from which the anatomically detailed computational models used in this study were developed. Segmentation was conducted semi-automatically in the open-source package Seg3D (www.seg3d.org) to delineate: blood pool, myocardium, infarct BZ and scar,^3^ in addition to the surrounding bath. Following up-sampling and smoothing, tetrahedral finite element meshes were created using CGAL library (https://www.cgal.org/) and smoothed using Meshtool^4,5^ creating meshes with average edge lengths of $330 \mu m \pm25 \mu m$ for myocardium, $600 \mu m \pm10 \mu m$ for the bath. Realistic myocardial fibre architecture was incorporated into the meshes using a rule-based approach.^6^

In four of the generated LV models, there was no visible critical isthmus (CI), known to be a requirement to sustain monomorphic VT. Consequently, in a similar manner in other studies^7^, these models were manually adjusted to include a CI. Specifically, a narrow and long channel (around 3mm in width) in the original scar was retagged to be Border zone to serve as CI based on the following restrictions:

- the channel should be along the fibre orientation, as often found clinically^8^.
- the size and shape of the channel should be qualitatively comparable to the other three porcine models
- the channel should be long enough to induce and sustain VTs where the wavelength, given by conduction velocity (CV) times the effective refractory period (close to action potential duration (APD)) is shorter than the length of conducting pathway.

2. Bidomain model

The electrophysiological model used here is the bidomain model^9^:

$$\nabla\cdot\left( \sigma_{i}\nabla\varphi_{i} \right)=\beta I_{m} (1)$$

$$-\nabla\cdot\left( \sigma_{e}\nabla\varphi_{e} \right)=\beta\left( I_{m}-I_{e} \right) (2)$$

where $\sigma_{i}$ and $\sigma_{e}$ are the intra- and extracellular conductivities, $\varphi_{i}$ and $\varphi_{e}$ are the intra- and extracellular electrical potentials, $\beta$ is the surface area of membrane contained within a unit

volume equal to ${0.14 \mu m}^{-1}$, $I_{m}$ is the transmembrane current density and $I_{e}$ is the extracellular stimulus current density. The transmembrane current density is given as:

$$I_{m}=C_{m}\frac{\partial V_{m}}{\partial t}+I_{ion}\left( V_{m},\eta\right) (3)$$

where $C_{m}$ is the membrane capacitance per unit area, $t$ is time and $I_{ion}$ is the ionic current density depending on $V_{m}$ and $\eta$ which is a vector of state variables describing channel gating

and ionic concentrations. The transmembrane potential $V_{m}$ is:

$$V_{m}=\varphi_{i}-\varphi_{e} (4)$$

The extracellular bath is described as:

$$\nabla\cdot\left( \sigma_{b}\nabla\varphi_{b} \right)=0 (5)$$

where $\sigma_{b}$ is the extracellular bath conductivity and $\varphi_{b}$ is the electrical potential in the bath space.

At the tissue boundaries, no flux condition is imposed for $\varphi_{i}$ while for extracellular potential $\varphi_{e}$ it is continuous as:

$$\boldsymbol{n}\cdot\left( \sigma_{b}\nabla\varphi_{b} \right)=\boldsymbol{n}\cdot\left( \sigma_{e}\nabla\varphi_{e} \right) (6)$$

$$\varphi_{b}=\varphi_{e} (7)$$

At the boundaries of the extracellular bath, no flux condition is imposed.

For computational efficiency, in certain circumstances the monodomain model was applied which is derived from the bidomain equations (assuming equal anisotropy ratios) as:

$$\nabla\cdot\left( \sigma_{m}\nabla V_{m} \right)=\beta\left( I_{m}-I_{s} \right) (8)$$

where $\sigma_{m}$ is the harmonic mean conductivity tensor.

Anisotropic conductivity was assigned to reproduce the characteristic of fibre structure. For the healthy tissue, the intracellular and extracellular conductivity used were $\sigma_{il}=0.174, \sigma_{it}=0.019, \sigma_{el}=0.625, \sigma_{et}=0.236 S/m$based on previous experiments.^10^ Based on our previous VT induction work,^11^ the conductivity of border zone (BZ) was set to 50% of the conductivity for healthy tissue to simulate the slowed conduction due to gap junction uncoupling and fibre disarray. The scar was assumed to be electrically insulating, with extra-cellular conductivity set to 0.05 S/m, based on previous works.^12,13^ The bath conductivity, representing the extra-cardiac bath space and intra-cavity blood pools was set to $1 S/m$.

3. VT induction

In order to induce stable, monomorphic episodes of VT, LV models were paced either at the base or apex. Firstly, prior to tissue-level pacing, cell models were preconditioned at the single-cell level, constituting 100 cycles at 2 Hz in order to produce stable single-cell states. These initial states were propagated to the tissue-level model and then paced with three stimulus S1 at a basic cycle length of 500 ms, followed by an S2 of a shorter coupling interval, successively reduced by 10 ms increments from 500 ms until unidirectional block was observed. If S2 capture failed, another premature stimulus S3 was added, following the same process until a block was observed. Note that for each model during the process of VT initiation, the ionic cell properties and conductivities in the BZ were assigned to be different from the healthy tissue to provide a substrate for VT-induction (i.e. uni-directional block). Different combinations of variations of ionic current conductance were used in the BZ, including 30%-65% reduction of the conductances of the potassium currents $I_{Kr}$ and $I_{Ks}$, 10%-90% reduction of the conductance of the sodium current $I_{Na}$ and 50% reduction of tissue conductivities, based on previous works^7,11,14,15^. Consequently, during VT initialization, each porcine model had different ionic property variations to one another. As shown previously^11^, such changes to the peri-infarct zone are a prerequisite in order to establish the necessary repolarization heterogeneity to initiate unidirectional block. Once unidirectional block had been successfully achieved, the state of the tissue was saved (checkpointed) for use in the next step.

In order to sustain the induced VTs, the ionic properties in the BZ were re-adjusted back to be the same as the healthy tissue.^11^ This was a required step in order to decrease the wavelength (within the BZ) to facilitate reentry, as also performed in previous work^7,11,16,17^. Note that the characteristic ‘zig-zag’ propagation in these infarcted regions^18,19^ may be explicitly represented by simulating patchy fibrosis within the BZ in the computational models, as shown in our previous work^11^. Such a change has the similar desired effect of achieving slowed conduction (necessary for re-entry sustenance) along with facilitating unidirectional block (due to electrotonic load mis-matches at fast pacing), without changing the ionic properties in BZ in mid-simulation. However, such detailed representations are often prohibited in large-scale models, due to their increased computational loads, as was the case here. In addition, following the simulation re-start, the conductivities of healthy tissue and BZ were adjusted, without altering their conduction velocities by more than 60%, similar to a previous study^7^. All of these changes were towards the goal of achieving a large number of VTs with different morphologies and cycle lengths. Following this change, all VTs were simulated for 2 seconds to ensure they were monomorphic and stable, giving one/two stable VT episodes for each of the pig models. States were then saved (checkpointed) for each pig model and used as the initiate states for next step.

In order to generate a number of VT episodes with various VT cycle lengths (VTCLs) and dynamics in each model, we adjusted conductivities and ionic properties in the BZ, as in the literature. ^7,11,14,15,17^ Specifically, the ionic properties in the BZ were altered by using different combinations of the below changes:

1. 30%-65% reduction of the conductances of the potassium currents $I_{Kr}$ and $I_{Ks}$
2. 10%-90% reduction of the conductance of the sodium current $I_{Na}$.

In some cases, the tissue conductivities were also adjusted slightly without varying conduction velocity of more than 10%, to prevent the VTs from self-termination. All VT episodes were then simulated for further 2 seconds to ensure stability of the MVT prior to applying electrotherapy.

In summary, these modifications resulted in 73 monomorphic, stable VTs generated in the 7 LV models using this induction protocol. Figure S1 shows the activation time maps of induced VTs in each individual porcine model.


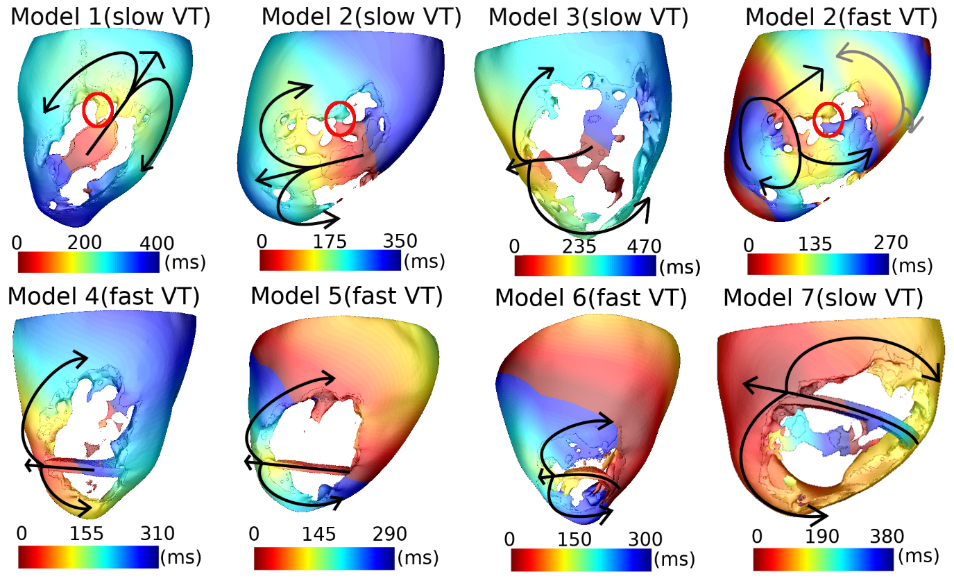


Figure S1: The activation time maps of three slow VTs and five fast VTs induced in the porcine models. The scar regions are removed in order to clearly show the propagation pathway of the activation wave. Both slow and fast VTs are induced in Model 2. The black arrows show the propagation direction of the VT. Red circles mark small pathways from the CI which may lead to alternate propagation circuits. In the fast VT in Model 2, extra grey arrows show the propagation direction from the previous VT cycle.

4. ATP delivery sites and extracellular potential recordings (EGMs) sensing sites

ATP can be delivered from standard ICDs and multipolar electrodes in CRT-D devices to certain specific areas. Here, we selected six delivery locations that mimic typical delivery locations in these devices. It consists of three bespoke ’scar-specific’ locations near the CI along the RV septum named as ”proximal” locations and three locations distal from the scar, replicating pacing delivered from CRT-Ds, utilising electrodes on leads in the coronary sinus. These six ATP delivery locations in the biventricular models are shown in Figure S2A. The ”*Entrance*” is located where a VT wavefront enters the CI, while “*Exit*” is where it exits. The “*CI*” is located in the middle of CI. In order to find realistic CRT-D pacing locations in all 7 LV models, a standardized universal ventricular coordinate (UVC) system was computed on all models^20^. Three common CRT-D pacing locations were chosen in all models using UVCs based on other study^21^. The three locations include “*Posterior*”, “*Lateral base*” and “*Lateral distal*” as shown in Figure S2A. Note that the additional anatomical representations of right ventricle as shown in Figure S2A are for illustrating the relative locations and orientation of the ATP delivery sites. The simulations were only applied to the LV models only. In Figure S2B, the averaged distance of the six pacing locations from the scar in seven models are shown. The three proximal locations have around $5 mm$ distance from the scar comparing to more than $30 mm$ in the three distal locations. Note that as shown in Figure S2, the average distance from LD location to the scar in all models ($33 mm$) is shorter than LB and Po locations ($38 and 53 mm$).

The standard ICDs and CRT-D devices can not only deliver electrotherapy but also work as sensing devices where the nearfield/far-field EGMs can be recorded. We recovered the EGMs during ATP application simulations using the commonly used $Phi\_e$ recovery method^22,23^ to obtain far-field extracellular potential recordings at a specific location using transmembrane potential data of the source (the myocardium) through an integral solution to Poisson’s equation^24^ , which assumes that the heart is embedded within an infinite conducting medium. Thus, only the specific (relative) locations of the sites at which extracellular potentials need to be recovered from are required (i.e. the can). The relative location of the can (and other recording sites) were obtained by transforming the hearts into a reference frame of a default torso, as shown in Figure S2C.

Five typical sensing points were chosen here which replicate sensing locations in ICDs and CRT-Ds. In order to find the relative locations of these five points, all LV geometries were registered to the LV enclosed in an existing human torso mesh using the LV long axis.^25^ Then the coordinates of the five sense points were derived accordingly as shown in Figure S2C. Note that similar to our previous EGM simulation studies ^26,27^, the electrode locations displaced a few millimetres away from the surface of cardiac tissue. Also, the sensing vectors are always constructed as bipolar recordings, i.e. the difference between two recording sites. Then five EGM sensing vectors were recorded during ATP application including: can to Superior vena cava (SVC) coil, can to RV ring, SVC coil to RV ring, RV tip to RV ring and LV coil at the lateral base to RV tip.


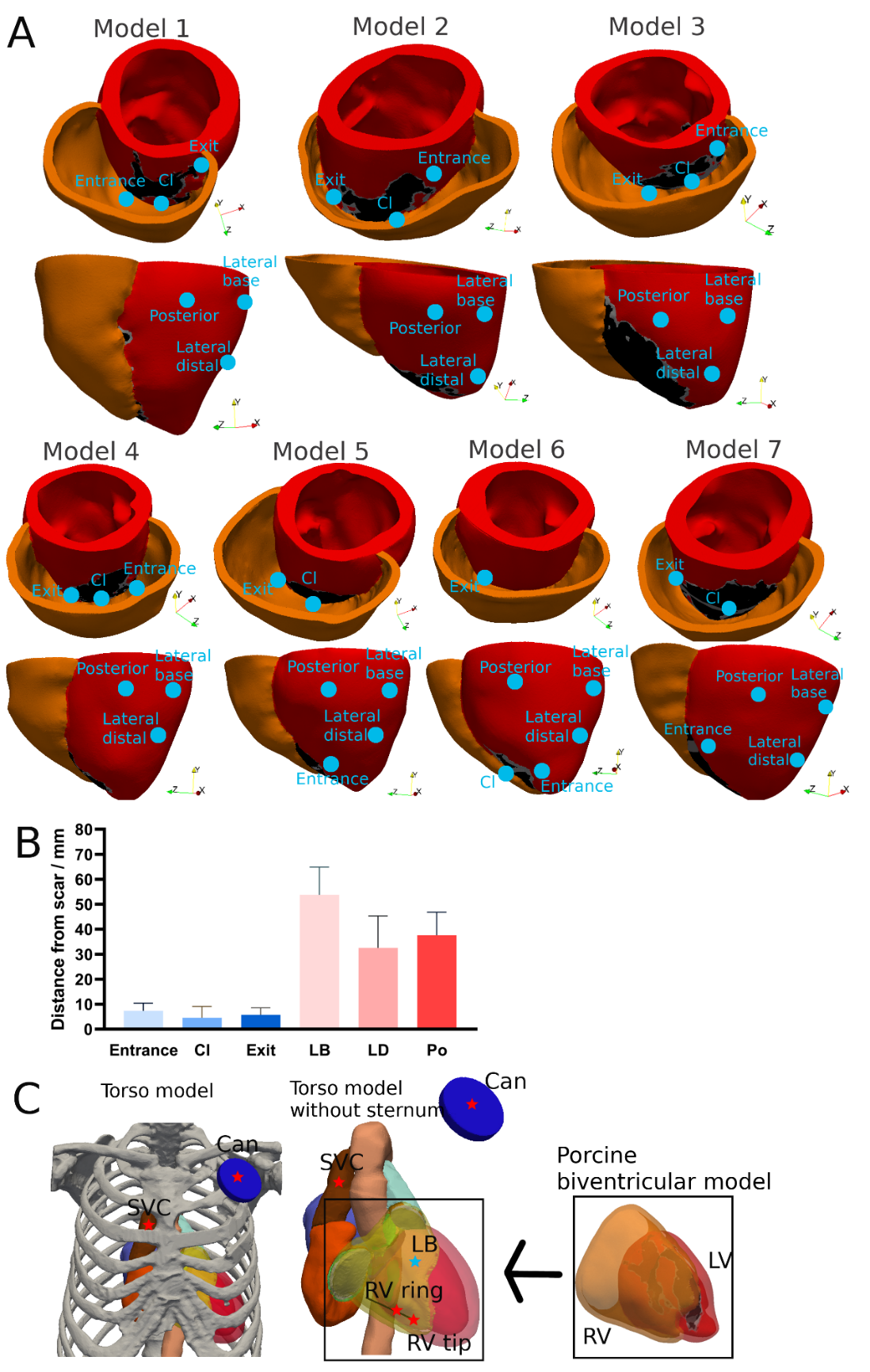


Figure S2: A. Seven porcine biventricular models showing the 6 proximal and distal locations in two views as in Figure 1B. Proximal locations are at RV-septum proximal to the scar and distal locations at the lateral LV which replicate CRT-D devices. B.The distances of all 6 pacing locations from the scar. C. Typical EGM sensing points in ICDs and CRT-Ds in a human torso model where the middle figure shows the torso without the rib cage. LV and RV are shown with partial opacity in pink and yellow. The right figure is the porcine biventricular model (right black box) which is rotated and moved to the same location of LV and RV as in the human torso model. The sensing locations include a can near the clavicle, Superior vena cava (SVC) coil, RV tip, RV ring and LV electrode at the lateral base (LB). LB location marked by a blue star is at the same location of the lateral base in Figure 1B.

5.Utility of our virtual ’library’ of simulated VT episodes

The VTs in this study were induced by similar protocols used in other computational modelling studies.^7,11^ Although VTCLs were seen to vary significantly between models, driven by the explicit infarct anatomy and CI, they were within the range of other recent works.^7,28^ Detailed analysis also showed how the complex structure of the scar and CI can be responsible for multiple possible re-entrant pathways, resulting in different VT dynamics which affect electrotherapy success, emphasising the importance of using high-resolution image-based models in this context. Furthermore, the overall efficacy (56%) in terminating all VTs using burst ATP electrotherapy with 0.23% acceleration in this study is comparable to clinical observations and other computational studies^7,29,30^ as is the improvement of efficacy with the application of an additional ATP sequence.^7,30,31^ In addition, we found that ATP was less effective on fast, compared to slow VTs, for all delivery locations (Figure [5A](https://emckclac-my.sharepoint.com/personal/k2031494_kcl_ac_uk/Documents/ATP_heartrhythm/QS_ATP_Supplementnew_MJB_QS_11Nov_2.docx#_bookmark7)), consistent with the literature.^29,30,32^ Overall, the analysis of our induced VTs underscore their clinical utility for comparing electrotherapy protocols.

6. Early termination detection algorithm (ETDA)

Here, we provide a detailed description of ETDA as shown in Figure 3. During the application of ATP, the EGMs starting from the application time of the pulse to just before the next pulse application were recorded. Then the correlation coefficient of two successive EGMs (corresponding to two successive pulses) were calculated. The correlation coefficient is defined as:

$$\rho\left( A,B \right)=\frac{1}{N-1}\sum_{i=1}^{N} \frac{{(A}_{i}-\bar{A}){(B}_{i}-\bar{B})}{\sigma_{A}\sigma_{B}} (9)$$

where $A=(u_{1},u_{2}, u_{3},\ldots u_{N})$ and $B=(v_{1},v_{2}, v_{3},\ldots v_{N})$ are two successive EGMs, $\bar{A}$ and $\bar{B}$ are their mean values and $\sigma_{A}$, $\sigma_{B}$ are their standard deviation. Five EGMs are recorded for each pulse, which were then averaged to be one correlation array as $\rho_{12}, \rho_{23},\ldots\rho_{78}$where for example, $\rho_{12}$ is the correlation coefficient of the 1st and 2nd EGMs pulses. The correlation coefficient can range from -1 to 1, where 1 represents the two successive EGMs that are highly correlated, i.e. they have similar EGM morphology. Therefore, if the correlation coefficients are seen to sharply drop, it suggests that the EGM morphologies vary greatly from pulse to pulse, which may indicate possible VT termination. We compared the averaged correlation arrays of all re-initiation cases with the actual VT termination time observed from simulation results. We found that there is a clear decreasing trend of the correlation coefficients before the time when the VT is terminated. Thus, we chose a threshold of the correlation coefficient as 0.16 to determine whether the VT is terminated or not, by the criteria of being able to detect the majority of VT termination in those re-initiation cases. Subsequently, the ETDA was applied to all ATP cases delivered from proximal (RV-Septum) locations and the new efficacy of ATP was calculated.

7. ATP efficacy comparison of applying 1 and 2 sequence

In order to investigate whether applying more sequences can improve ATP efficacy, we applied one or two ATP sequences and compared their efficacy on terminating VTs, as shown in Figure S3. Here, we see that ATP efficacy is improved to 56% at proximal locations and 58% at distal locations for 2 sequences, compared to 34% and 26% for 1 sequence of ATP delivery. The reasons for two sequences increasing ATP efficacy were not straight forward to assess in our analysis, but appeared to be a combination of additional pulses (increasing the chances of fully blocking the isthmus) and decreased cycle length compared with previous sequence (early capturing and propagation into larger area that may result in blockage).
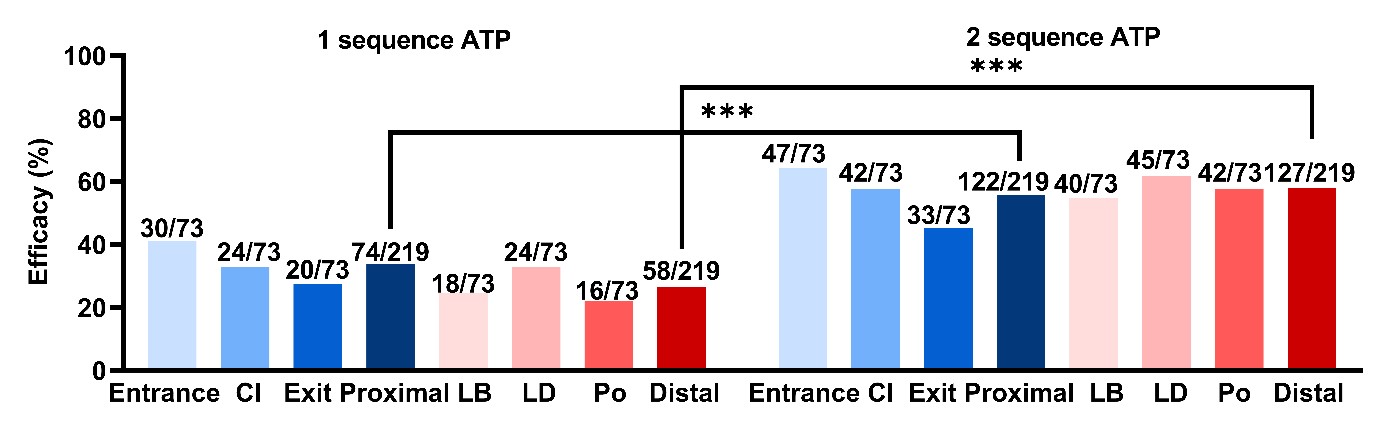


Figure S3: The efficacy of 1 sequence and 2 sequence ATP from 6 delivery locations including proximal locations (Entrance, CI and Exit) and distal locations (Lateral base, Lateral distal and Posterior). Shown also is the total efficacy of all proximal locations and all distal locations. Individual groups were compared using the Chi-square test, ***p<0.0001.

8.Additional analysis on Efficacy of ATP For Different VT Rates at Specific Delivery Locations

The efficacy of ATP varies for different delivery locations when separately analysed for slow vs fast VTs. As shown in Figure 5B, further detailed analysis on the efficacy for slow vs fast VT for distal locations reveals that the efficacy delivered from two specific distal locations: LB and Po, for slow VTs ($71\%$ and $73\%$) are significantly higher than for fast VTs ($44\%$ and $48\%$) (both $P=0.002$). However, the efficacy when delivering from the other distal location: LD on slow VTs ($71\%$) is also higher than on fast VTs ($64\%$), but with less significance ($P=0.07$).

9. Mechanism of Failed ATPs

As shown in Figure 5B, the majority of ATP cases failed due to insufficient prematurity. Here we show an example of ATP failed due to insufficient prematurity comparing with ATP that succeeds in Figure S4. As is shown in Figure S4A, the ATP is sufficiently aggressive that the excitable gap is reduced; although the orthodromic paced wavefront can ’follow’ the VT, entering the CI, when it tries to exit (where electronic loading is highest and propagation most vulnerable), it fails and terminates. However, in Figure S4B the pacing is not early enough; consequently, the orthodromic paced wave can follow the VT and also escape the isthmus.


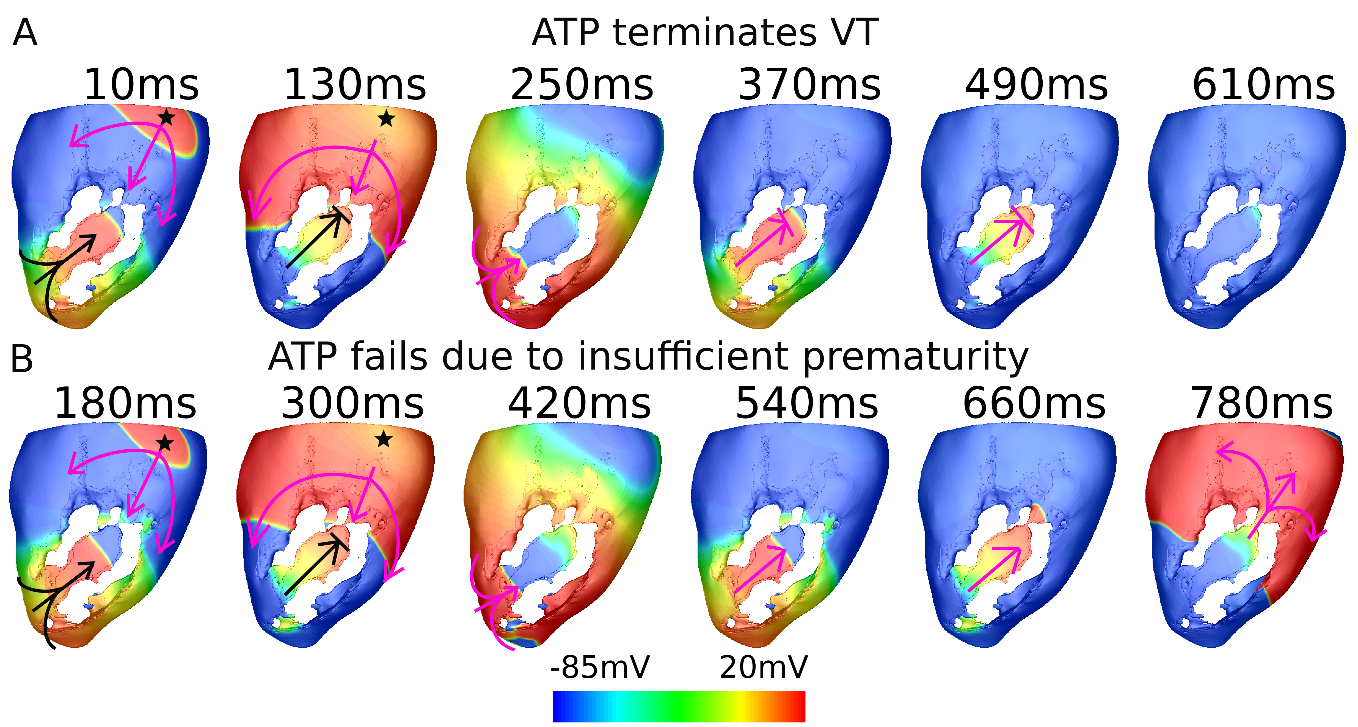


Figure S4: Example of successful and failed ATP. V m maps at different times during ATP in Model 1. Panel A shows an example of VT successfully terminated by ATP, with panel B showing another VT episode not terminated by ATP due to insufficient prematurity. Black stars show the pacing location. Black arrows show VT propagation and pink arrows show the ATP propagation. The last pulse in the 2nd sequence of ATP at time 10 ms in A and time 180 ms in B are presented. Black arrows show the propagation of the VT circuit, with pink arrows showing the propagation of the orthodromic and antidromic wavefronts from the applied ATP. Solid lines show areas of conduction block.

The least frequent mechanism of failed ATP (acceleration) only appears when pacing at the CI in Model 2, as shown in Figure 5(b) in the main paper. The only case of acceleration seen is shown in Figure S5. Similar to the slow VT of Model 2 (as shown in Figure S1), the original VT is blocked at the pathway which is marked with the black circle. The orthodromic wavefront from the applied ATP results in earlier depolarization of this blocked pathway from the CI, subsequently causing it to repolarize earlier, too. Thus, in the next cycle of the VT, the depolarization wave (of the VT) can propagate through this recovered pathway as it now regained its excitability. Because of this pathway, the total length of the original VT path was reduced, resulting in acceleration. The new VTCL is 300 ms, much smaller than 350 ms for the original VT.


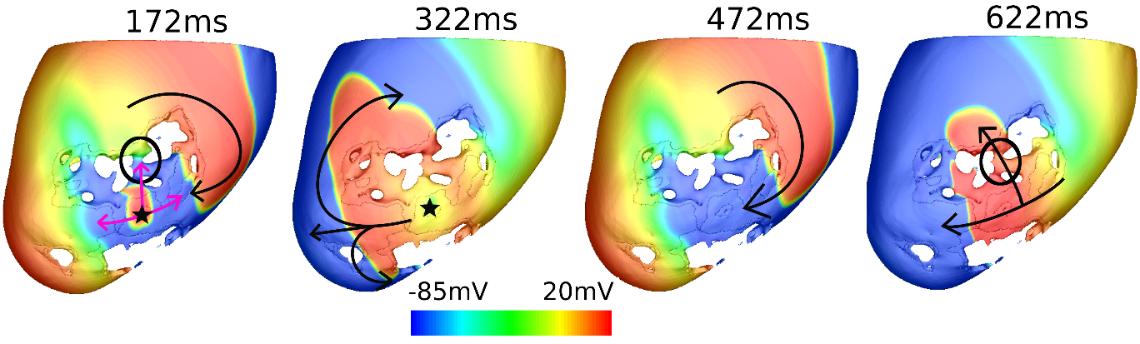


Figure S5: Vm maps at different times of failed ATP applying at CI in model 2 due to acceleration. The last pulse of ATP is applied at time 172 ms. Black stars show the pacing location. Black arrows show VT propagation and pink arrows show the ATP propagation. The shorter pathway from CI marked in black circles is blocked at 172 ms and then unblocked at 622 ms after ATP applies which accelerates the VT.

10. Assessment of ETDA performance

The performance of ETDA on these cases are summarised in Figure 7C. ATP cases (prior to/in absence of ETDA) are separately analysed as (1) VT-terminated, (2) VT not terminated, (3) re-initiation. The outcomes of ETDA can be summarised in four types. EDTA correctly identifies the right timing at which VT is terminated (blue); EDTA identified termination, but does so either earlier (orange) or later (grey) than the true termination time; EDTA fails to detect termination at all (yellow). Overall, ETDA correctly identifies the time of termination (or no termination for not terminated cases) in 68% of cases.

For not-terminated cases, ATP efficacy will not be changed for both detecting as “no termination” (69%) and “terminated too early” (31%). For terminated cases, ATP efficacy also will not be changed if it detects at the right timing (68%), does not detect (24%) or detects later than actual VT termination time (6%). However, 2 terminated cases (2%) were detected too early, which will reduce ATP efficacy. In the cases of re-initiation, 79% are detected at the right timing, causing an improvement in ATP efficacy. 21% of cases are either not detected by ETDA or detected too late, which will not change ATP efficacy. However, depending on the outcome of the full sequence of ATP (in absence of ETDA), the specific type of failure by ETDA may or may not impact ATP efficacy.

11. Mechanistic insights of ATP terminating VTs and implications

As shown in Figure 5A and B, the termination of VT using ATP is highly dependent on the VTCL and the delivery location. Therefore, we analysed the mechanism of ATP terminating VTs based on simulation results. Similar to the other studies reported,^33,34^ we observed that termination of VTs happens because the VT wavefront collided with the antidromic portion of the ATP wavefront and the orthodromic portion of ATP wavefront which blocked in the VT's refractory region (closing down the excitable gap). We found that VT termination tended to be more prevalent when pacing proximal to the scar, specifically within the CI location. This is because both antidromic and orthodromic ATP wavefronts are very close to or even in the excitable gap of the VT, which thus ensures penetration of the re-entrant circuit, resulting in more efficient termination. However, it is also important to note that pacing proximal to the scar also showed a high risk of re-initiation, as after the VT is terminated by the ATP, the successive ATP pulses result in antidromic wavefronts which expand, pulse by pulse, and finally form unidirectional conduction block and initiate functional re-entry in a reversed direction, as shown in Figure 6. Should ATP failure due to re-initiation be eliminated, for example through our ETDA approach, targeting the CI itself could improve ATP efficacy.

12. Separate analysis of ATP efficacy for different VT rates only on three realistic infarcted models

In this section, we analysed the ATP efficacy for different VT rates on three realistic infarcted models: Model 1, 2 and 3. Similar to Figure 5A and B, ATP efficacy were plotted as shown in Figure S6. As shown in Figure S6A, similar as in Figure 5, the ATP efficacy on slow VTs is significantly higher than on fast VTs (69% vs 23%, P<0.00001). Separate analysis on slow VTs shows that pacing distally is more effective than proximally (75% vs 63%, P=0.09), while this trend is reversely for analysing fast VTs (17% vs 30%, P=0.22). This is consistent with the results on the whole cohort as shown in Figure 5A. In this smaller cohort, ATP efficacy on different locations for different VT rates are compared in Figure S6B. For slow VTs and pacing proximally, their efficacy ranges from 56% to 78% which is comparable to 46%-73% in the whole cohort. Significant differences can be seen when comparing the ATP efficacy on slow and fast VTs at the locations entrance (78% vs 30%, P=0.0069), LB (74% vs 20%, P=0.0029), LD (74% vs 10%, P=0.00048) and Po (78% vs 20%, P=0.0013). Overall, this sub-cohort analysis shows similar overall trends in the data (albeit without reaching statistical significance) to the total cohort analysis on all 7 porcine models presented in the main manuscript: specifically, for slow VTs, pacing distally is more effective than pacing proximally, however for fast VTs, pacing proximally is actually more efficacious.


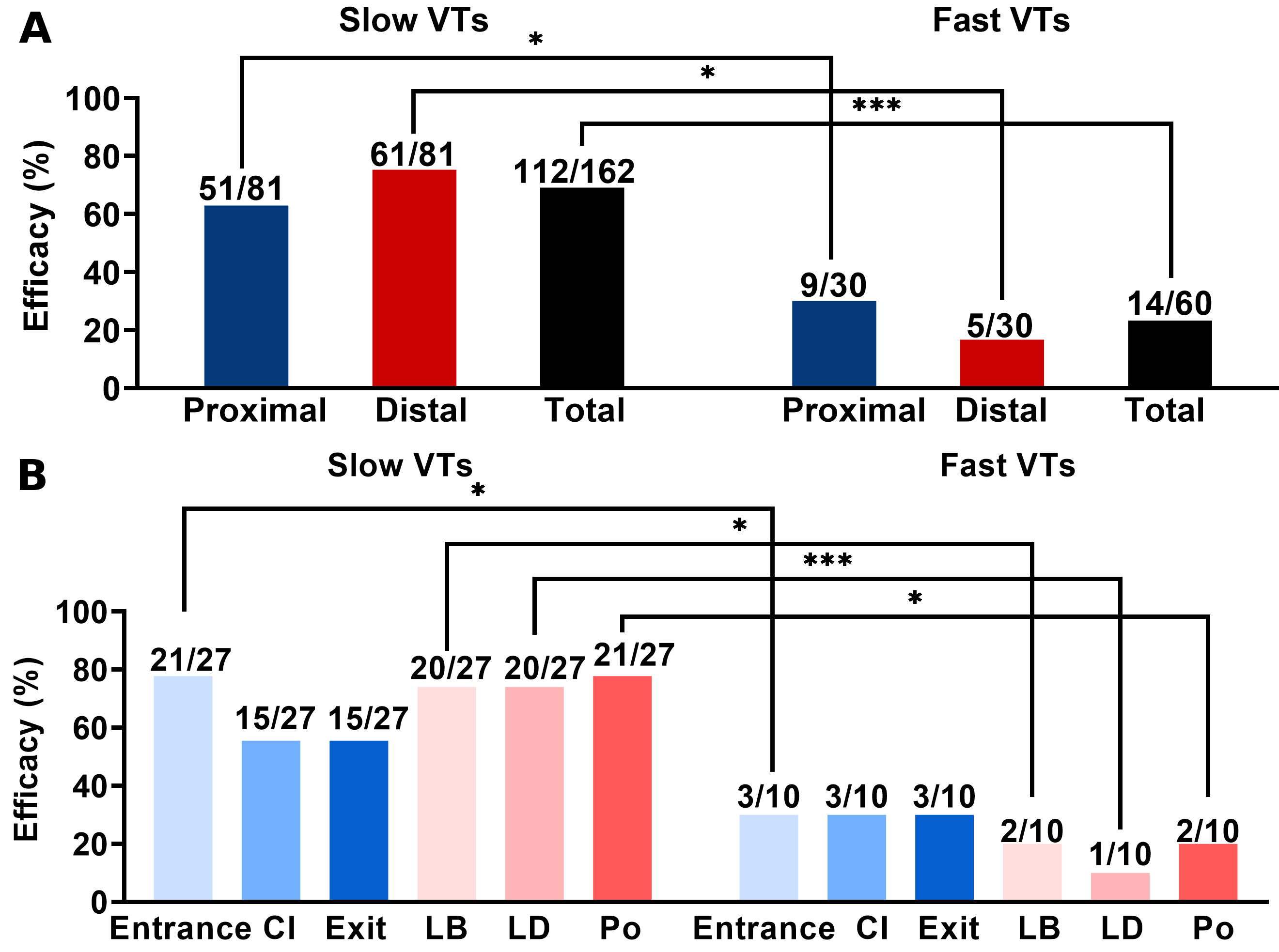


Figure S6: ATP efficacy assessment on three realistic infarcted models (Model 1,2 and 3) regarding VT rates and ATP failures. A: Efficacy of ATP proximal and distal delivery and the total for fast and slow VTs. B: ATP efficacy of all locations. Location nomenclature as Figure 4. *p<0.05, ***p<0.0001.

References

1. Tschabrunn CM, Roujol S, Nezafat R, et al.: A swine model of infarct-related reentrant ventricular tachycardia: Electroanatomic, magnetic resonance, and histopathological characterization. Heart Rhythm [Internet] Elsevier, 2016; 13:262–273. Available from: http://dx.doi.org/10.1016/j.hrthm.2015.07.030

2. Whitaker J, Neji R, Kim S, et al.: Late Gadolinium Enhancement Cardiovascular Magnetic Resonance Assessment of Substrate for Ventricular Tachycardia With Hemodynamic Compromise. Front Cardiovasc Med [Internet] 2021; 8:744779. Available from: http://www.ncbi.nlm.nih.gov/pubmed/34765656%0Ahttp://www.pubmedcentral.nih.gov/articlerender.fcgi?artid=PMC8576410

3. Whitaker J, Neji R, Byrne N, et al.: Improved co-registration of ex-vivo and in-vivo cardiovascular magnetic resonance images using heart-specific flexible 3D printed acrylic scaffold combined with non-rigid registration. Journal of Cardiovascular Magnetic Resonance Journal of Cardiovascular Magnetic Resonance, 2019; 21:1–15.

4. Neic A, Gsell MAF, Karabelas E, Prassl AJ, Plank G: Automating image-based mesh generation and manipulation tasks in cardiac modeling workflows using Meshtool. SoftwareX Elsevier B.V., 2020; 11:100454.

5. Campos FO, Orini M, Arnold R, et al.: Assessing the ability of substrate mapping techniques to guide ventricular tachycardia ablation using computational modelling. Computers in Biology and Medicine Elsevier Ltd, 2021; 130:104214.

6. Bayer JD, Blake RC, Plank G, Trayanova NA: A novel rule-based algorithm for assigning myocardial fiber orientation to computational heart models. Annals of Biomedical Engineering 2012; 40:2243–2254.

7. Swenson DJ, Taepke RT, Blauer JJE, et al.: Direct comparison of a novel antitachycardia pacing algorithm against present methods using virtual patient modeling. Heart Rhythm [Internet] Elsevier Inc., 2020; 17:1602–1608. Available from: https://doi.org/10.1016/j.hrthm.2020.05.009

8. Chillou C De, Magnin-Poull I, Andronache M, et al.: Showing up channels for postinfarct ventricular tachycardia ablation. PACE - Pacing and Clinical Electrophysiology. 2012, pp. 897–904.

9. Henriquez CS: Simulating the electrical behavior of cardiac tissue using the bidomain model. Crit Rev Biomed Eng 1993; 21(1):1–77.

10. Clerc L: Directional differences of impulse spread in trabecular muscle from mammalian heart. The Journal of Physiology 1976; 255:335–346.

11. Campos FO, Whitaker J, Neji R, et al.: Factors Promoting Conduction Slowing as Substrates for Block and Reentry in Infarcted Hearts. Biophysical Journal [Internet] 2019; 117:2361–2374. Available from: http://www.sciencedirect.com/science/article/pii/S0006349519306757

12. Connolly AJ, Bishop MJ: Computational Representations of Myocardial Infarct Scars and Implications for Arrhythmogenesis. Clinical Medicine Insights: Cardiology 2016; 10s1:27–40.

13. Colli-Franzone P, Gionti V, Pavarino LF, Scacchi S, Storti C: Role of infarct scar dimensions, border zone repolarization properties and anisotropy in the origin and maintenance of cardiac reentry. Mathematical Biosciences Elsevier, 2019; 315:108228.

14. Prakosa A, Arevalo HJ, Deng D, et al.: Personalized virtual-heart technology for guiding the ablation of infarct-related ventricular tachycardia. Nature Biomedical Engineering Nature Publishing Group, 2018; 2:732–740.

15. Arevalo HJ, Vadakkumpadan F, Guallar E, et al.: Arrhythmia risk stratification of patients after myocardial infarction using personalized heart models. Nature Communications Nature Publishing Group, 2016; 7.

16. Pashakhanloo F, Herzka DA, Halperin H, McVeigh ER, Trayanova NA: Role of 3-Dimensional Architecture of Scar and Surviving Tissue in Ventricular Tachycardia: Insights from High-Resolution Ex Vivo Porcine Models. Circulation: Arrhythmia and Electrophysiology [Internet] 2018; 11. Available from: http://ahajournals.org

17. Qian S, Connolly A, Mendonca-Costa C, et al.: An in-silico assessment of efficacy of two novel intra-cardiac electrode configurations versus traditional anti-tachycardia pacing therapy for terminating sustained ventricular tachycardia. Computers in Biology and Medicine [Internet] Elsevier Ltd, 2021; 139:104987. Available from: https://doi.org/10.1016/j.compbiomed.2021.104987

18. de Bakker JMT, van Capelle FJL, Janse MJ, et al.: Slow conduction in the infarcted human heart: “Zigzag” course of activation [Internet]. Circulation. 1993. Available from: http://ahajournals.org

19. de Baker JMT, Coronel R, Tasseron S, et al.: Ventricular tachyrdia in the infarcted, Langendorff-perfused human heart: Role of the arrangement of surviving cardiac fibers. J Am Coll Cardiol 1990; 15:1594–1607.

20. Bayer J, Prassl AJ, Pashaei A, et al.: Universal ventricular coordinates: A generic framework for describing position within the heart and transferring data. Medical Image Analysis [Internet] Elsevier B.V., 2018; 45:83–93. Available from: https://doi.org/10.1016/j.media.2018.01.005

21. Antoniadis AP, Behar JM, Sieniewicz B, Gould J, Niederer S, Rinaldi CA: A comparison of the different features of quadripolar left ventricular pacing leads to deliver cardiac resynchronization therapy. Expert Review of Medical Devices Taylor & Francis, 2017; 14:697–706.

22. Neic A, Campos FO, Prassl AJ, et al.: Efficient computation of electrograms and ECGs in human whole heart simulations using a reaction-eikonal model. Journal of Computational Physics [Internet] Elsevier Inc., 2017; 346:191–211. Available from: http://dx.doi.org/10.1016/j.jcp.2017.06.020

23. Plonsey R, Barr C. R: Bioelectricity A Quantitative Approach. New York, NY: Springer 2002; .

24. Plonsey R: Bioelectric sources arising in excitable fibers (Alza lecture). Annals of Biomedical Engineering 1988; 16:519–546.

25. Strocchi M, Lee AWC, Neic A, et al.: His-bundle and left bundle pacing with optimized atrioventricular delay achieve superior electrical synchrony over endocardial and epicardial pacing in left bundle branch block patients. Heart Rhythm [Internet] Elsevier Inc., 2020; 17:1922–1929. Available from: https://doi.org/10.1016/j.hrthm.2020.06.028

26. Monaci S, Gillette K, Puyol-Antón E, et al.: Automated Localization of Focal Ventricular Tachycardia From Simulated Implanted Device Electrograms: A Combined Physics–AI Approach. Frontiers in Physiology 2021; 12:1–15.

27. Monaci S, Strocchi M, Rodero C, et al.: In-silico pace-mapping using a detailed whole torso model and implanted electronic device electrograms for more efficient ablation planning. Computers in Biology and Medicine [Internet] Elsevier Ltd, 2020; 125:104005. Available from: https://doi.org/10.1016/j.compbiomed.2020.104005

28. Cossú S: Resynchronization Therapy During Sustained Ventricular Tachycardia. Journal of Innovations in Cardiac Rhythm Management 2017; 8:2590–2593.

29. Sweeney MO: Antitachycardia pacing for ventricular tachycardia using implantable cardioverter defibrillators: Substrates, methods, and clinical experience. PACE - Pacing and Clinical Electrophysiology 2004; 27:1292–1305.

30. Santini M, Lunati M, Defaye P, et al.: Prospective multicenter randomized trial of fast ventricular tachycardia termination by prolonged versus conventional anti-tachyarrhythmia burst pacing in implantable cardioverter-defibrillator patients-Atp deliVery for pAiNless ICD thErapy (ADVANCE-D) tr. Journal of Interventional Cardiac Electrophysiology 2010; 27:127–135.

31. Anguera I, Dallaglio P, Martínez-Ferrer J, et al.: Shock Reduction with Multiple Bursts of Antitachycardia Pacing Therapies to Treat Fast Ventricular Tachyarrhythmias in Patients with Implantable Cardioverter Defibrillators: A Multicenter Study. Journal of Cardiovascular Electrophysiology 2015; 26:774–782.

32. Gulizia MM, Piraino L, Scherillo M, et al.: A randomized study to compare ramp versus burst antitachycardia pacing therapies to treat fast ventricular tachyarrhythmias in patients with implantable cardioverter defibrillators: The PITAGORA ICD trial. Circulation: Arrhythmia and Electrophysiology 2009; 2:146–153.

33. Byrd IA, Rogers JM, Smith WM, Pollard AE: Comparison of conventional and biventricular antitachycardia pacing in a geometrically realistic model of the rabbit ventricle. Journal of Cardiovascular Electrophysiology 2004; 15:1066–1077.

34. Byrd IA, Kay MW, Pollard AE: Interactions between paced wavefronts and monomorphic ventricular tachycardia: Implications for antitachycardia pacing. Journal of Cardiovascular Electrophysiology 2006; 17:1129–1139.
